# Poly(lactic acid)/Zinc/Alginate Complex Material: Preparation and Antimicrobial Properties

**DOI:** 10.3390/antibiotics10111327

**Published:** 2021-10-30

**Authors:** Marcin H. Kudzin, Małgorzata Giełdowska, Zdzisława Mrozińska, Maciej Boguń

**Affiliations:** Lukasiewicz Research Network-Textile Research Institute, Brzezinska 5/15, 92-103 Lodz, Poland; malgorzata.gieldowska@iw.lukasiewicz.gov.pl (M.G.); zdzislawa.mrozinska@iw.lukasiewicz.gov.pl (Z.M.); maciej.bogun@iw.lukasiewicz.gov.pl (M.B.)

**Keywords:** alginate, alginic acid, antibacterial activity, biodegradable composite, composite, melt-blown, nonwoven fabric, polymer, poly(lactide) PLA, zinc(II)chloride

## Abstract

The aim of this study was to investigate an antimicrobial and degradable composite material consisting of melt-blown poly(lactic acid) nonwoven fabrics, alginate, and zinc. This paper describes the method of preparation and the characterization of the physicochemical and antimicrobial properties of the new fibrous composite material. The procedure consists of fabrication of nonwoven fabric and two steps of dip-coating modification: (1) impregnation of nonwoven samples in the solution of alginic sodium salt and (2) immersion in a solution of zinc (II) chloride. The characterization and analysis of new material included scanning electron microscopy (SEM), specific surface area (SSA), and total/average pore volume (BET). The polylactide/alginate/Zn fibrous composite were subjected to microbial activity tests against colonies of Gram-positive (*Staphylococcus aureus*), Gram-negative (*Escherichia coli*) bacterial strains, and the following fungal strains: *Aspergillus niger* van Tieghem and *Chaetomium globosum*. These results lay a technical foundation for the development and potential application of new composite as an antibacterial/antifungal material in biomedical areas.

## 1. Introduction

Wound healing is a dynamic and complex process (phases: hemostasis, inflammation, proliferation, and maturation) affected by several factors, which needs an appropriate surrounding to achieve accelerated healing [1]. Modern wound healing dressing should exhibit non-toxic and non-allergenic properties, be capable of maintaining high humidity at the wound site while removing excess exudate, antibacterial characteristics or at least impermeability to bacteria, enable gaseous exchange, and be cost effective [2,3,4,5]. These requirements are partly fulfilled by the physico-chemical properties of PLA (biocompatibility, biodegradability, mechanical strength) [6,7,8,9,10,11,12,13,14,15] and to a much larger extent by PLA-composites equipped with a wide spectrum of antimicrobial agents, including inorganic microbials [16,17].

Among the various inorganic bactericides applied in antibacterial polymers [18,19,20,21,22,23,24,25,26,27,28,29,30,31,32,33,34,35,36,37] increasing attention has focused on zinc salts [38], which is vitally essential for many biological processes [39], toxic to microbials and nontoxic to higher organisms [40], and a cheap and antibacterially efficient [41,42,43,44,45,46,47] inorganic antimicrobial. The zinc biochemistry and biology are determined by the complexation and hydration of zinc ions [48,49,50,51,52,53,54]. The zinc (II) coordination environment (tetrahedral vs. octahedral zinc complexes [55]) is limited in proteins to oxygen, nitrogen, and sulfur donors from the side chains of a few amino acids [31,33]. In living organisms, usually tetracoordinated Zn(II) is redox-inert since its standard reduction potential is negative (Zn^2+^(aq) + 2e^−^ → Zn(s); Eo = −0.76V) [56].

Since an effective antibacterial PLA composite should exhibit prolonged antibacterial activity, a stable surface deposition/attachment of zinc to PLA presents a major problem. Due to the low affinity of metallic cations to carboxylic ester bonds [57], PLA weakly binds zinc ions, and its antibacterial PLA-Zn^(2+)^ composites require an interface covering layer with high affinity to copper. Such requirements fulfill alginates, biodegradable biopolymers [58,59,60,61,62,63] applied as a basis for drug delivery [64,65,66,67,68,69,70,71,72,73,74,75,76,77,78], tissue engineering [79,80,81,82,83,84,85,86,87,88,89], and wound dressings [90,91,92,93,94,95,96,97,98].

The role of alginate in antibacterial finishing of textiles was reviewed recently by Li et al. [99]. PLA-ALG have been investigated in a few papers for various applications [100,101,102,103,104,105,106,107].

The strong affinity of alginates to metal cations allows their application as antibacterial hybrids (e.g., [108,109,110,111]). ALG-Zn^(2+)^ complexes have been described in 792 papers [112] (e.g., [113,114,115,116,117,118,119,120,121,122,123,124]).

**Figure 1:**
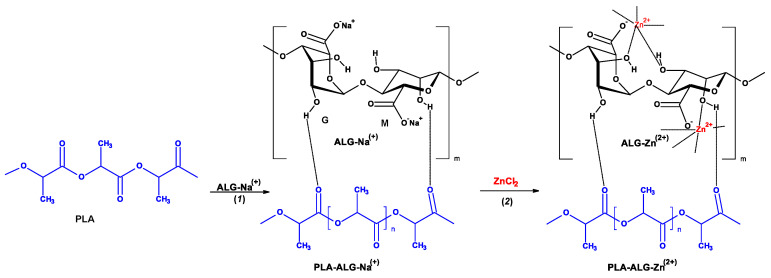
The reactions involved in the preparation of fibrous composite: PLA→PLA-ALG-Na^(+)^ →PLA-ALG-Zn^(2+)^. The structure of alginate is presented as a linear copolymer –[GM]n- with homopolymeric blocks of (1-4)-linked β-D-mannuronate (M) and its C-5 epimer α-L-guluronate (G) residues.

**Figure 2:**
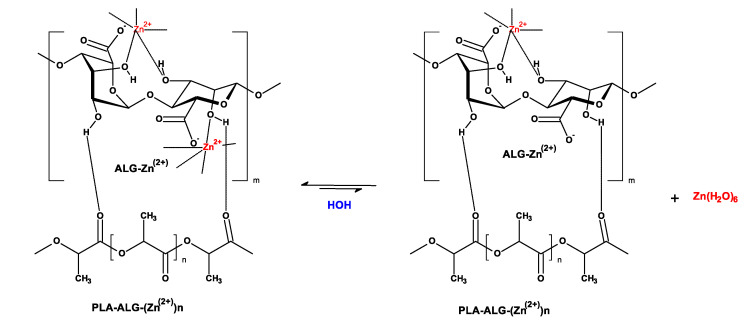
The reactions involved in the release of zinc ion from the PLA-ALG-Zn^(2+)^ composite in an aqueous environment.

As part of our investigations focused on the functionalization of textile materials [125,126,127,128,129,130,131], we propose the use of an alginate film covering the PLA matrix (PLA-ALG), which, after adding copper salts, was cross-linked to form an outer space coating with strongly bound copper ions (Figure 1). Such a PLA-Alg-Zn^(2+)^ composite slowly releases zinc ions, ensuring its long-lasting antibacterial activity (Figure 2).

## 2. Materials and Methods

### 2.1. Materials

- Alginic acid sodium salt, C5H7O4COONa (CAS: 9005-38-3) were purchased from Millipore Sigma (St. Louis, MO, USA);
- Bacterial strains: *Escherchia coli* (ATCC 25922) and *Staphylococcus aureus* (ATCC 6538) were purchased from Microbiologics (St. Cloud, MN, USA);
- Fungal strains: *Aspergillus niger* van Tieghem (ATCC 6275) and *Chaetomium globosum* (ATCC 6205) were purchased from Microbiologics.
- Poly(lactic acid) was provided by NatureWorks LLC (Minnetonka, MN, USA), type Ingeo™ 3251D, with an MFR value of 30–40 g/10 min (at 190 °C/2.16 kg);
- Zinc (II) chloride, ZnCl2, 98% (CAS: 7646-85-7) was purchased from Millipore Sigma.

### 2.2. Methods

#### 2.2.1. Preparation of Fibrous Material—Melt-Blown Process

The poly(lactic acid) melt-blown nonwoven fabrics with a basis weight of 250 g/m^2^ were produced by using a one-screw laboratory extruder (Axon, Limmared, Sweden). The extruder head has 30 holes with a 0.25 mm die orifice diameter each. The polylactide (PLA) granulate for melt blowing was dried at 80 °C to constant weight. The process parameters of melt blowing are given in Table 1.

#### 2.2.2. Modification—Dip-Coting

The PLA melt-blown nonwoven fabrics samples were modified by dip-coting, a two-step method: (1) impregnation in the solution of alginic acid sodium salt and (2) immersion in the solution of zinc (II) chloride. Samples of PLA nonwoven fabric were impregnated in a homogeneously dispersed polysaccharide solution (0.5%) for 1 min (sample: PLA-Alg-Na^(+)^), then each sample was immediately transferred into two different aqueous solutions of zinc (II) chloride and re-immersed for 1 min (sample PLA-Alg-Zn^(2+)^-1 re-immersed in 5% ZnCl_2_ solution and sample PLA-Alg-Zn^(2+)^-2 re-immersed in 10% ZnCl_2_ solution). Then, the samples were squeezed and dried for 5 h at 50 °C to constant weight. The modifier components are given in Table 2.

#### 2.2.3. Morphological and Structural Characterization—Scanning Electron Microscopy

Examination of the microscopic structures was carried out using a HITACHI S-4700 scanning electron microscope equipped with a Thermo NORAN EDS X-ray microanalyzer. The topographic analysis of the tested samples was carried out in low vacuum with a beam energy of 10 kV and at magnifications of 800× and 1600×.

#### 2.2.4. Morphological and Structural Characterization—Specific Surface Area

The specific surface area was determined by the Brunauer, Emmet, and Teller method (BET). Measurements were carried out on an Autosorb-1 apparatus (Quantachrome Instruments, Boynton Beach, FL, USA), using nitrogen as a sorption agent and an adsorption isotherm at 77 K. In each experiment, approximately 1–2 g of a given sample were weighed and used. Prior to the analysis, the samples were dried in 105 °C for 24 h and degassed at room temperature. Measurements were made in duplicate, and the results were presented as a mean value.

#### 2.2.5. Morphological and Structural Characterization—Contact Angle and Wettability

Surface wettability was determined by static measurements of the water wetting angle. The wetting angle was measured by the “sitting droplet” method using a drop shape analysis (DSA) system DSA 10 Mk2 (Kruss GmbH, Hamburg, Germany). Water drops of 0.25 µL were applied to each clean and dry sample. The apparent contact angle was calculated as the average of 10 measurements [132].

#### 2.2.6. Chemical Characterization—Atomic Absorption Spectrometry with Flame Excitation

**Figure 3:**
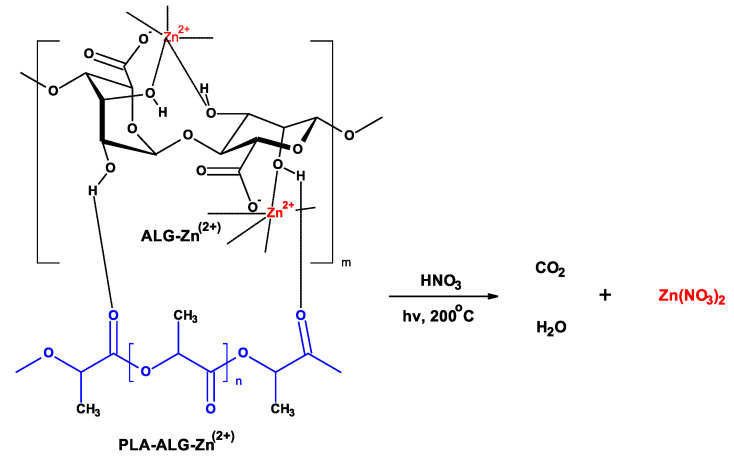
Mineralization of PLA-Alg-Zn^(2+)^.

Determination of the zinc content in PLA-Alg-Zn^(2+)^ composites was assessed by the FAAS method. The previous sample was mineralized (Figure 3) using a single-module Magnum II microwave mineralizer from Ertec (Wroclaw, Poland), in similar way as was described earlier [133].

Determination of the zinc (II) ions was performed by atomic absorption spectrometry with flame excitation using a Thermo Scientific Thermo Solar M6 (LabWrench, Midland, ON, Canada) spectrometer equipped in similar way as was described earlier [133,134]. Measurements were made in triplicate and the results were presented as a mean value.

#### 2.2.7. Antimicrobial Activity

The antibacterial activity of PLA-Alg-Zn^(2+)^ material was assessed according to standard PN-EN ISO 20645:2006 [135] against a representative colony of Gram-negative and Gram-positive bacteria (*Escherchia coli*/*Staphylococcus aureus*). The antifungal activity of composites was tested according to PN–EN 14119:2005 [136] against an *Aspergillus niger* van Tieghem (ATCC 6275) and *Chaetomium globosum* (ATCC 6205). All tests (modified samples and unmodified PLA) were carried out in duplicate.

## 3. Results and Discussion

### 3.1. Scanning Electron Microscopy

The presented SEM images illustrate changes in the morphology of the fiber surface of the investigated samples. These changes are the result of modification of PLA nonwoven fabric with sodium salt solution of alginic acid and ZnCl_2_.

**Figure 4:**
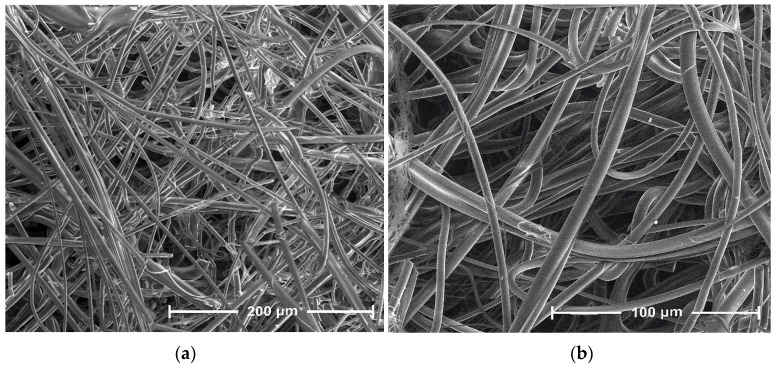
SEM images of PLA, magnification: (**a**) 800× and (**b**) 1600×.

**Figure 5:**
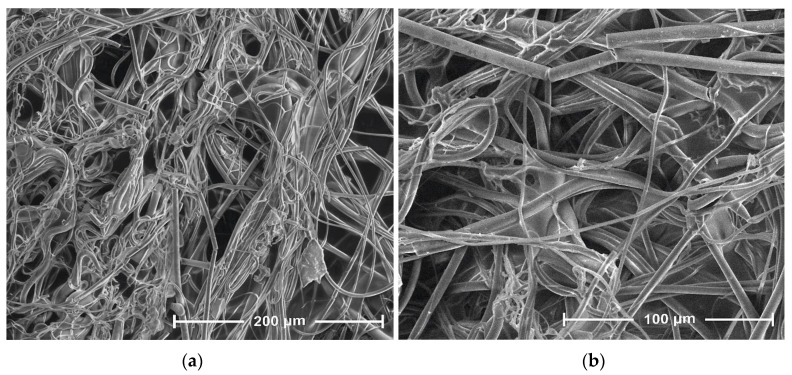
SEM images of PLA-ALG-Na^(+)^, magnification: (**a**) 800× and (**b**) 1600×.

**Figure 6:**
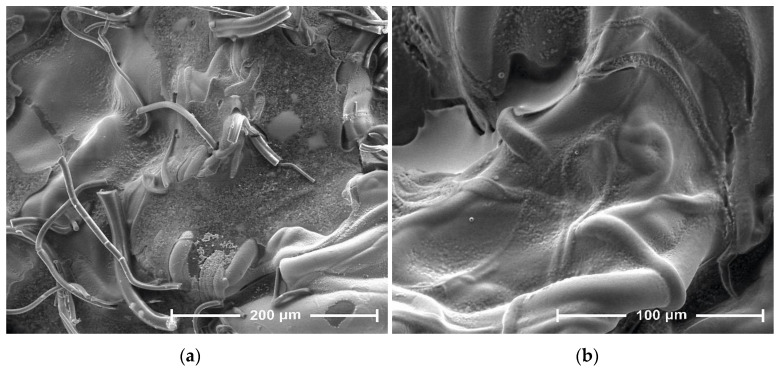
SEM images of PLA-ALG-Zn^(2+)^, magnification: (**a**) 800× and (**b**) 1600×.

The SEM image of the unmodified nonwoven fabric presents a mesh of randomly oriented fibers with interconnected pores and a comparatively smooth surface (Figure 4). Modification of the nonwoven fabric with sodium alginate solution (PLA→PLA-ALG^(−)^ Na^(+)^) induced the formation of a film on the fiber surface, during which the PLA fibrous structure serves as a matrix for a deposited alginate, presumably by the formation of hydrogen bonds between the HO group of alginate and the carbonyls of the carboester functions of PLA. This modification slightly affected the overall morphology of the formed composite surface, maintaining the fibrous structure of the PLA→PLA-ALG^(−)^Na^(+)^ composites but with more irregular shapes and less visible pores. Additionally, agglomerates of sodium alginate (ALG-Na^(+)^) can be seen on single fibers of PLA-ALG^-^Na^(+)^ (Figure 5). Further modification of the temporary composite PLA-ALG^(−)^Na^(+)^ with ZnCl_2_, (PLA-ALG^(−)^ Na^(+)^→PLA-ALG-Zn^(2+)^) led to immense changes in its morphology, probably due to the formation of interchain complexes PLA^1^-ALG^1^-Zn^(2+)^-ALG^2^-PLA^2^ (PLA^1^-ALG^1(−)^Na^(+)^→PLA^1^-ALG^1^-Zn^(2+)^→PLA^1^-ALG^1^-Zn^(2+)^-ALG^2^-PLA^2^). Therefore, the fibrous structure of the resulting composite was largely deformed with an increase of surface roughness, with multiple zinc agglomerates (Figure 6), resembling a polymer film, with integrated PLA fibers.

**Figure 7:**
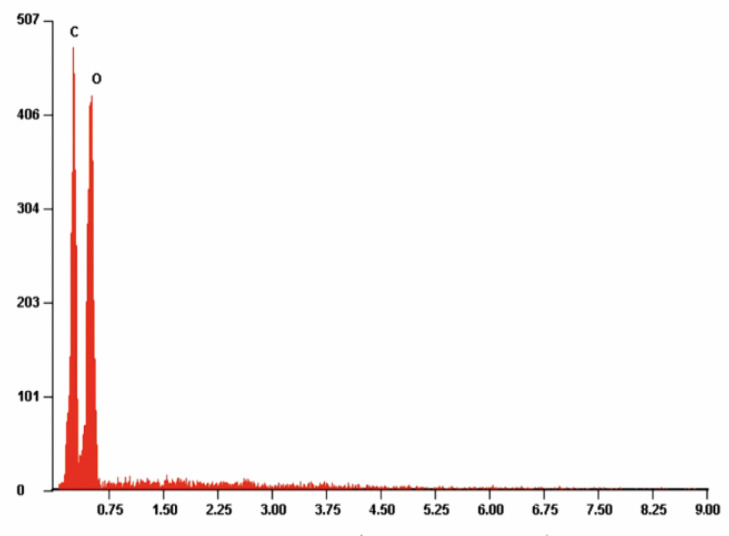
Example of the energy-dispersive X-ray spectroscopy (EDS) spectrum of PLA nonwoven fabric.

**Figure 8:**
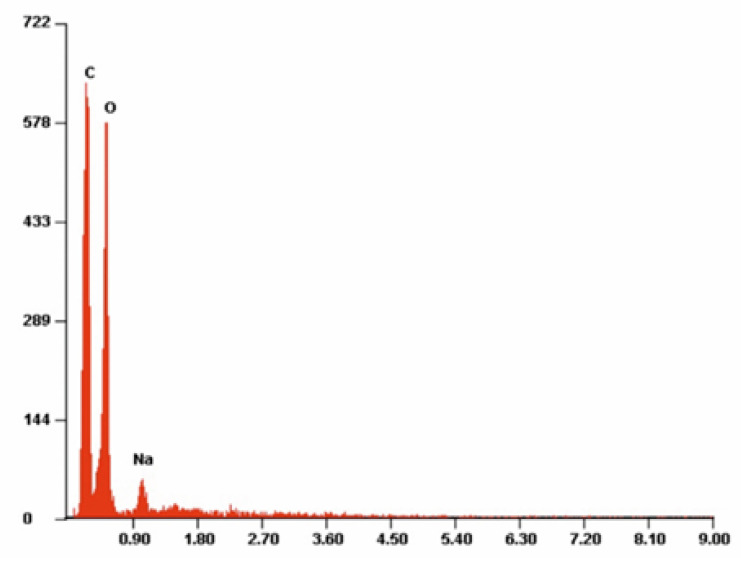
Example of the energy-dispersive X-ray spectroscopy (EDS) spectrum of PLA-ALG-Na^(+)^.

**Figure 9:**
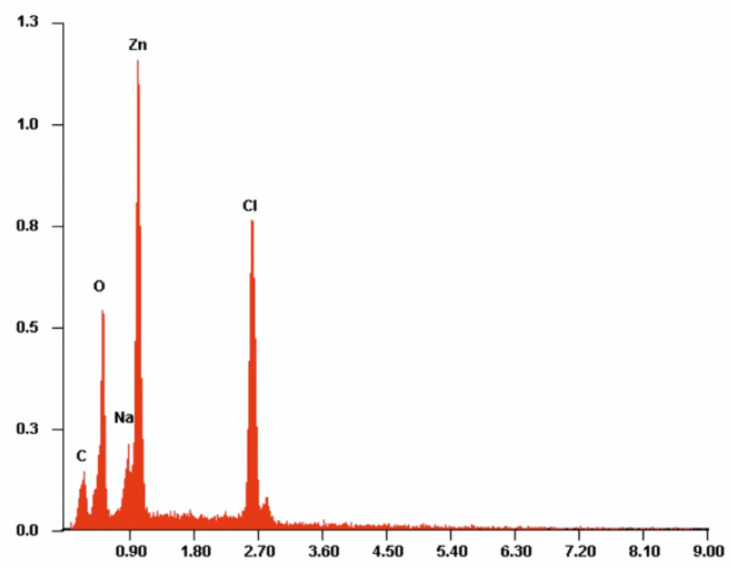
Example of the energy-dispersive X-ray spectroscopy (EDS) spectrum of PLA-ALG-Zn^(2+)^.

Figure 7, Figure 8 and Figure 9 present example of the EDS spectra for PLA, PLA-Alg-Na^(+)^, and PLA-Alg-Zn^(2+)^ (10%) samples (EDS data are presented as a plot of the peak intensity versus energy (keV)). Table 3 shows the chemical composition of the tested materials, obtained by quantitative analysis using EDS X-ray microanalysis. The table presents the averaged results obtained from five measurement points.

When analyzing the spectrum in Figure 7, it can be observed that the sample contains elements characteristic for polylactide, that is, carbon and oxygen. Whereas the PLA-ALG-Na^(+)^ sample, because of the modification with sodium alginate, is characterized by the presence of an additional peak, derived from Na atoms (Figure 8). Additionally, modification of PLA-ALG-Na^(+)^ nonwoven fabric with zinc (II) chloride contributed to the presence of further peaks, originating from zinc and chloride. The presence of chloride is confirmed by the content of NaCl in the samples.

Quantitative EDS analysis showed that modification of PLA nonwoven fabric with sodium alginate did not significantly change the carbon and oxygen contents of the samples. Slight decreases in the content of these elements were observed: about 0.28 at. % and about 1.38 at. % for carbon and oxygen, respectively. Additionally, small concentrations of sodium (1.65 at %) were noted for the PLA-ALG-Na^(+)^ sample. The EDS results for the samples modified with ZnCl_2_ show that the content of Zn and Cl in the samples increased with the increasing chloride concentration. However, because it showed the best antibacterial and antifungal properties, the results are presented for the PLA-ALG-Zn^(2+)^ sample (10%). We can observe that because of the rapid cross-linking of the sample surface and the formation of a kind of film on the surface, the sodium content increased twice, compared to the PLA-ALG-Na^(+)^ sample. On the other hand, the high content of zinc (14.22%) confirms that the agglomerates shown in the photos are agglomerates of a divalent metal (Figure 9). Moreover, it can be seen that when the samples were modified with ZnCl_2_, the carbon and oxygen content decreased significantly (by 23.39 at. % and 11.26 at. %, respectively). A high value of standard deviation indicates an irregular distribution of the chemical components.

### 3.2. Specific Surface Area

The specific surface area [m^2^/g] and total pore volume [cm^3^/g] of the composites are presented in Table 4.

The results of the specific surface area and the total pore volume measurements correspond to our previous studies [111]. The modification of poly(lactic acid) (PLA) nonwoven fabric with alginate (PLA-Alg-Na^(+)^) and zinc (II) chloride (PLA-Alg-Zn^(2+)^-1/PLA-Alg-Zn^(2+)^-2) leads to significant growth of the specific surface area (BET). The BET of the poly(lactic acid) sample was equal to 0.221 [m^2^/g]. The impregnation of unmodified PLA nonwoven fabric in the solution of alginic acid sodium salt resulted in an increase in the value of the specific surface area (0.58 [m^2^/g]). The dip-coating two-step modification, i.e., impregnation in a solution of alginic acid sodium salt and immersion in a ZnCl_2_ solution (5%/10%), caused an even further increase in BET to 0.5211 and 0.8331 m^2^/g, respectively. The increase of the observed specific surface area for the modified samples (PLA-Alg-Na^(+)^/PLA-Alg-Zn^(2+)^-1/PLA-Alg-Zn^(2+)^-2) may be related with their higher mesoporosity. Higher mesoporosity was confirmed by the total pore volume, which increased from 9.102 × 10^−4^ cm^3^/g for the PLA nonwoven fabric to 1.64 × 10^−3^ cm^3^/g for PLA-Alg-Na^(+)^, 2.750 × 10^−3^ cm^3^/g for PLA-Alg-Zn^(2+)^-1, and 3.093 × 10^−3^ cm^3^/g for PLA-Alg-Zn^(2+)^-2.

### 3.3. Contact Angle and Wettability

**Figure 10:**
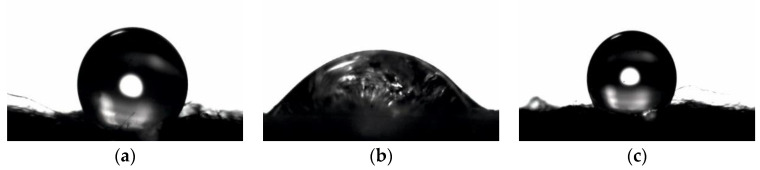
Contact angle images for: (**a**)—PLA, (**b**)—PLA-ALG-Na^(+)^, (**c**)—PLA-ALG-Zn^(2+)^.

The analysis of the contact angle enabled an assessment of the wettability of the surfaces of the tested materials. The results demonstrated that PLA and PLA-ALG Zn^(2+)^ nonwoven fabrics were characterized by a hydrophobic character of the surface, for which the average contact angle was 122.79° and 114.98°, respectively (Figure 10a,c). In contrast, the modification with sodium alginate changed the character of the material surface, which became hydrophilic with an average contact angle of 45.67° (Figure 10b).

**Figure 11:**
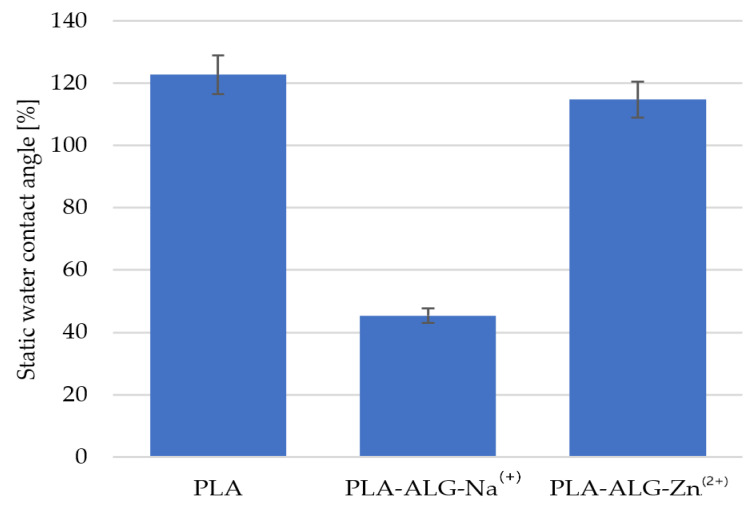
The static water contact angle of PLA and its composites.

As shown in Figure 10 and Figure 11, modification of PLA nonwoven fabric with sodium alginate significantly changed the nature of the nonwoven fabric surface from hydrophobic to hydrophilic. This is due to the presence of the Na^+^ ions contained in alginate, which make it soluble in water. On the other hand, another modification with ZnCl_2_ resulted in substitution of sodium ions with divalent metal ions (Zn^2+^), thanks to which the crosslinking process occurred on the surface and alginate covering fibers became insoluble in water. This changed the nature of the nonwoven surface from hydrophilic to hydrophobic again. However, it is characterized by lower surface hydrophobicity than the PLA nonwoven fabric, due to the Na^+^ ions present on the surface in small amounts, which was confirmed by SEM-EDS analysis.

### 3.4. Flame Atomic Absorption Spectrometry

Determination of the metal concentration in the PLA-Alg-Zn^(2+)^ samples was assessed by the FAAS spectrometry, and the results are shown in Table 5.

The results of the determination of the zinc concentration in PLA-Alg-Zn^(2+)^ samples show that the metal content in complex materials depends on the variant of applied modifier dip-coating solution and type of solution of Zinc (II) chloride (Table 5). The higher concentration of the used zinc (II) chloride solutions (10%) resulted in the higher content of the metal on PLA-Alg-Zn^(2+)^-2 material (39.71 g/kg), and the lower concentration of zinc (II) chloride (5%) gave a relatively lower content of Zn in the sample (PLA-Alg-Zn^(2+)^-1—11.55 g/kg). Additionally, the FAAS spectrometry measurements also indicate that the distribution of zinc in the material is quite uniform (approximately 7.5%).

### 3.5. Antimicrobial Activity—Disc-Diffusion Assay

**Figure 12:**
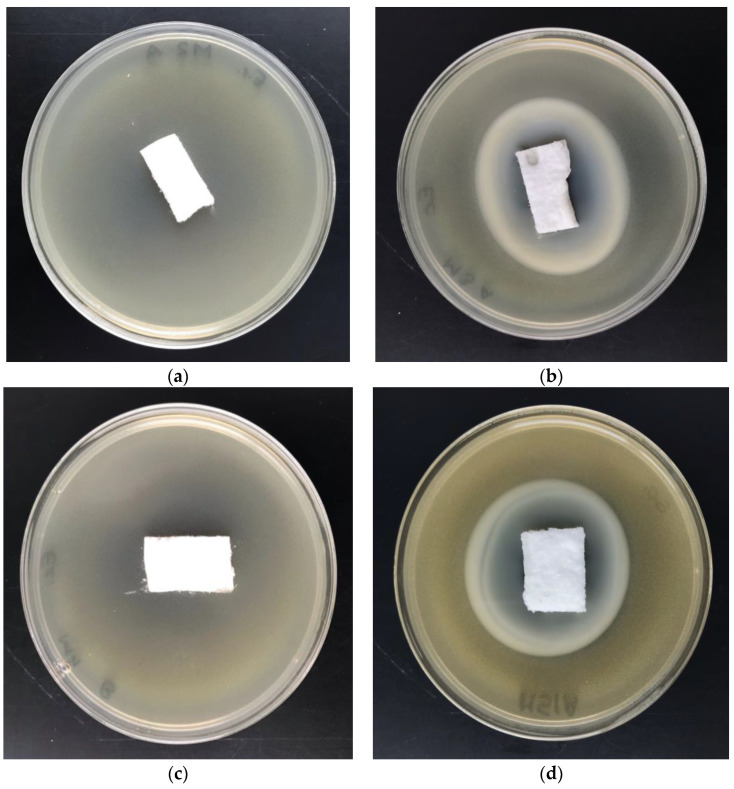
Zone of inhibition test results on Petri dishes showing antibacterial activity: (**a**,**b**)—*E. coli*; (**c**,**d**)—*S. aureus*; (**a**,**c**) Control: unmodified PLA nonwoven fabric, (**b**,**d**) Sample: PLA-ALG-Zn^(2+)^.

**Figure 13:**
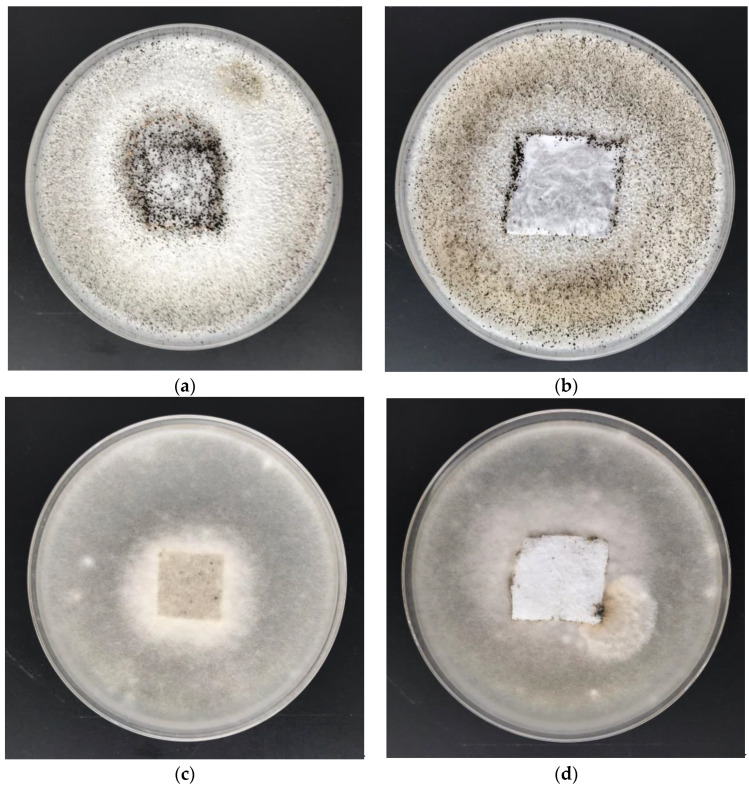
Zone of inhibition test results on Petri dishes showing antifungal activity: (**a**,**b**)—*A. niger*; (**c**,**d**)—*C. globosum*; (**a**,**c**)—Control: unmodified PLA nonwoven fabric; (**b**,**d**)—Sample: PLA-ALG-Zn^(2+)^ material.

The antimicrobial activity of PLA-Alg-Zn^(2+)^ composites was investigated by the disk diffusion method using Gram-negative (*E. coli*) and Gram-positive (*S. aureus*) bacteria (Table 6, Figure 12) and representative fungus species: *Aspergillus niger* and *Chaetomium globosum* (Table 7, Figure 13).

Control samples as unmodified PLA and modified by alginic acid sodium salt material (PLA-Alg-Na^(+)^) exhibited strong growth of bacterial and fungal colonies covering the entire surface of the samples placed on Petri dishes (Figure 12a,c and Figure 13a,c). Poly(lactic acid) material functionalized with a zinc/alginate complex, regardless of the metal concentration in PLA-Alg-Zn^(2+)^ composites in the range from 11 to 39 [g/kg] (Table 5), showed an inhibitory effect against *S. aureus* bacteria and fungus species expressed by zones of inhibition and no visible growth on/under the samples (Table 6 and Table 7). The increase of the zinc concentration in PLA-Alg-Zn^(2+)^ composites caused an increase of the antimicrobial properties of the material. The PLA-Alg-Zn^(2+)^-2 (39 [g/kg] of Zn) sample showed antimicrobial properties for all variants of the tested microorganisms (Figure 12b,d and Figure 13b,d). The results obtained in accordance with the EN-ISO 20645:2006 and EN 14119:2005 standards confirm the antimicrobial protection of PLA-Alg-Zn^(2+)^ complexes against various microorganisms [135,136].

## 4. Conclusions

In this study, we developed and characterized a complex material consisting of alginic acid sodium salt, poly(lactide), and zinc (II) chloride. PLA-ALG-Zn^(2+)^ composite was fabricated from biodegradable PLA polymer by the melt-blown method, and then the obtained fibrous material was modified by dip-coating, using alginic acid sodium salt and zinc (II) chloride. Structural characterization of the new material was achieved by scanning electron microscopy (SEM), and determination of the specific surface area and wettability. The chemical compositions of the PLA-Alg-Zn^(2+)^ composites were identified using energy-dispersive X-ray spectroscopy EDS (C, O, Zn surface analysis) and atomic absorption spectrometry with flame excitation (Zn content in bulk). The obtained complex material exhibited an antimicrobial in vitro action against representative bacteria: *Escherichia coli* and *Staphylococcus aureus*, and fungus species: *Chaetomium globosum* and *Aspergillus niger*. From the point of view of our previous work, these results are promising regarding the material’s applicability in different fields of materials science in the medical or healthcare industry.

## Figures and Tables

**Table 1:** Processing parameters of the melt blown process.

Parameter	Value
Extruder screw zone temperatures	195–260 °C
Extruder head temperature	260 °C
Air temperature	260 °C
Air flow rate	8 m^3^/h
The area density of nonwoven fabric	250 g/m^2^

**Table 2:** Composition of the dip-coating solution of poly(lactide) (PLA) surface modifier (%).

Sample Assignments/Name	Mixture Components of Film-Forming Material (%)		
	Sodium Alginate Solution	Zinc (II) Chloride Solutions	
	0.5%	5%	10%
PLA	−	−	−
PLA-Alg-Na^(+)^	+	−	−
PLA-Alg-Zn^(2+)^-1	+	+	−
PLA-Alg-Zn^(2+)^-2	+	−	+

**Table 3:** Quantitative results of the EDS analysis of PLA, PLA-ALG-Na^(+)^, and PLA-ALG-Zn^(2+)^.

**PLA**					
Atom	C	O			
At. %	54.70	45.30			
Std. dev.	0.18	0.18			
Wt. %	47.55	52.46			
Std. dev.	0.18	0.18			
**PLA-ALG-Na^(+)^**					
Atom	C	O	Na		
At. %	54.42	43.93	1.65		
Std. dev.	1.61	2.55	0.03		
Wt. %	45.45	52.09	2.47		
Std. dev.	0.54	0.13	0.29		
**PLA-ALG-Zn^(2+)^**					
Atom	C	O	Na	Zn	Cl
At. %	31.31	34.05	3.42	14.22	17.01
Std. dev.	1.21	3.58	0.76	0.52	1.29
Wt. %	14.62	21.18	4.61	36.14	23.45
Std. dev.	2.08	2.38	0.88	1.52	4.13

**Table 4:** The specific surface area and total pore volume for unmodified PLA nonwoven and PLA-ALG-Zn^(2+)^ composites.

Sample Name	Specific Surface Area [m^2^/g]	Total Pore Volume [cm^3^/g]
PLA	0.221 ± 0.03	9.10 × 10^−^^4^
PLA-Alg-Na^(+^^)^	0.587 ± 0.03	1.64 × 10^−3^
PLA-Alg-Zn^(2+)^-1	0.521 ± 0.02	2.75 × 10^−^^3^
PLA-Alg-Zn^(2+)^-2	0.833 ± 0.03	3.09 × 10^−3^

**Table 5:** Zinc concentration in PLA-Alg-Zn^(2+)^ samples.

Sample	Zn Concentration [g/kg]
PLA	0.003
PLA-Alg-Na^(+)^	0.003
PLA-Alg-Zn^(2+)^-1	11.55
PLA-Alg-Zn^(2+)^-2	39.71

**Table 6:** Antibacterial activity results according to standards EN-ISO 20645:2006 of PLA-Alg-Zn^(2+)^ composites [135].

Sample	Average Inhibition Zone (mm)	
	*E. coli*	*S. aureus*
PLA nonwoven	0	0
PLA-Alg-Na^(+)^	0	0
PLA-Alg-Zn^(2+)^-1	0	>1
PLA-Alg-Zn^(2+)^-2	>1	>1
Concentration of inoculum [CFU/mL]: *E. Coli*—1.8 × 10^8^, *S. Aureus*—1.6 × 10^8^		

**Table 7:** Antifungal activity results according to standards EN 14119:2005 of PLA-Alg-Zn^(2+)^ composites [136].

Sample	Average Inhibition Zone (mm)		Visual Evaluation
	*A. niger*	*C. globosum*	
PLA nonwoven	0	0	Visible, strong growth on/under the sample
PLA-Alg-Na^(+)^			
PLA-Alg-Zn^(2+)^-1	<1	<1	No visible growth on/under the sample
PLA-Alg-Zn^(2+)^-2			
Concentration of inoculum [CFU/mL]: *A. niger*—2.8 × 10^6^, *C. globosum*—2.2 × 10^6^			
